# A silicon microneedle array atmospheric pressure plasma ionization source for real-time trace gas chemical analysis

**DOI:** 10.1038/s41378-026-01291-4

**Published:** 2026-05-21

**Authors:** Bradley S. Chew, Dylan T. Koch, Patrick Gibson, Mitchell M. McCartney, Eva Borras, Nicholas J. Kenyon, Cristina E. Davis

**Affiliations:** 1 Department of Mechanical and Aerospace Engineering, One Shields Avenue, University of California, Davis, CA USA; 2 UC Davis Lung Center, Davis, CA USA; 3 Department of Electrical and Computer Engineering, One Shields Avenue, University of California, Davis, CA USA; 4 Department of Internal Medicine, UC Davis, Sacramento, CA USA

**Keywords:** Electrical and electronic engineering, Sensors

## Abstract

We have engineered, fabricated, and qualified ZAPPI (Zonal Atmospheric Pressure Plasma Ionizer), an array of hollow electrical discharge emitter micro needles for volatile organic compound ionization and detection. The device is optimized for portable applications requiring low power and trace analyte abundance. The 250 µm interelectrode gap requires just 1.1 mW, detecting analytes at $\dot{m}=23{ng}{s}^{-1}$ mass flow rates. We further demonstrate ZAPPI’s efficacy by ionizing several compounds representing different point of detection applications. These compounds include DMMP a simulated chemical warfare nerve agent, 2-butanone a widely studied biomarker in exhaled breath, Methyl salicylate a flavoring agent, and Naphthalene a toxic polycyclic aromatic hydrocarbon by-product in combustion. The wafer level fabrication of the ZAPPI device opens future possibilities for highly integrated trace gas detection lab on chip systems.

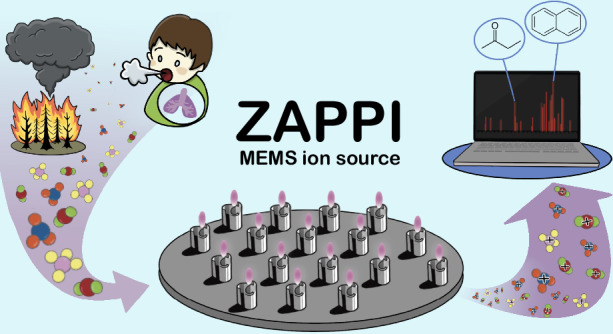

## Introduction

Chemical detectors are particularly interesting to the transducers and microelectromechanical (MEMS) communities. A confluence of factors, including interest in remote sensing, mature fabrication technologies ported from the integrated circuit sector, and advances in data processing present numerous scientific and commercial opportunities. Point-of-care health monitoring^1–4^, precision agriculture^5^, and air quality monitoring^6,7^ are key application areas poised to benefit from the development of a specific class of chemical sensors, trace gas analyzers. Exhaled breath metabolomic analysis represents one leading-edge investigation area in human health research and point-of-care diagnostics, immediately benefitted from the advancements in high-performance chemical sensors. In this particular use case, selectivity and sensitivity improvements will enable sensors to better detect minute metabolomic signal differences leading to better health outcomes.

However, existing chemical sensor technologies fall short, particularly in achieving sufficient sensitivity and chemical selectivity for point-of-sampling/point-of-need environments, or when continuous spatial-temporal data adds vital context to conclusions^8^. While the gold-standard trace gas analysis technique, mass spectrometry (MS), offers unmatched analytical power, its size, cost, and ancillary equipment typically preclude it from many field settings^9^. On the other hand, several other MEMS-based chemical sensors have reached commercial maturity^6^. Metal oxide sensors are small and inexpensive enough to be ubiquitously found in household electronics, from air quality monitors to vacuum cleaners. Although compact, sensors in this family near universally suffer from insufficient sensitivity in the ppb range and equally poor chemical selectivity. Ion mobility spectrometry (IMS) techniques bridge this gap by offering a more sensitive and selective atmospheric pressure trace gas analysis technique with significant inroads to miniaturization already having been made^10,11^.

Like many gas-phase chemical sensors, IMS operates by first ionizing neutral molecules into charged species, which are then detected and analyzed. While highly effective, radioactive ionization sources (e.g., Ni-63, Am-241) pose regulatory and procurement concerns, limiting widespread adoption of devices containing them. Ultraviolet ionization is another common ionization technique, but lacks efficiency converting electrical power to ion current and a low ionization energy threshold, typically below 12 eV^12^. Electrical discharge sources offer several advantages. By igniting a plasma from a neutral gas, typically nitrogen or helium, trace water vapor in the sample is decomposed providing the proton necessary to cluster with and ionize analytes^13^. Plasma ionization techniques generate high ion currents and ionization energies without the regulatory drawbacks imposed by a radioactive source. Additionally, electrical and carrier gas parameters can be changed to alter functional properties or improve efficiency^14,15^. Most germane to this work, electrical ionization sources can be miniaturized with MEMS manufacturing processes, allowing for down the line monolithic fabrication with other trace gas analyzer elements for a streamlined total detection system fabricated on-chip.

In this work, we describe the ideation, engineering design, fabrication, testing, and application of an atmospheric pressure plasma assisted microneedle ionization source. Plasma ionization sources have long been described in literature, most notably by Spindt et al. in his application of miniature volcano needle array^16^ for mass spectrometry ionization. Spindt describes a highly functional MEMS ionization array for chemical detection. He also details that after an errant vacuum leak the device ceased functionality after over 7 years, highlighting the environmental harshness and engineering challenges behind an atmospheric pressure ionization array. Since Spindt’s original publication many others have described atmospheric plasma ionization sources, and others have miniaturized them using microfabrication techniques^17^. While gas chromatography^18^, sample preparation^19,20^ and ion filtering^10^ have all been miniaturized and characterized, ionization sources have lagged in developing microfabricated alternatives. The presented work brings ionization sources to the level of these other components by describing the first silicon microfabricated atmospheric pressure plasma assisted ionization source verified with gold-standard mass spectrometry and integrated in line with a portable ion detector.

## Results and discussion

### Design and manufacture

The ZAPPI, Zonal Atmospheric Pressure Plasma Ionizer was designed for a simple purpose, to generate stable ionized species from neutral gas molecules and deliver ions to a downstream detector for chemical analysis. In brief, ZAPPI devices generate a spatially confined microfluidic atmospheric plasma zone, ionizing analytes passing through the hollow microfluidic needle device. A carrier gas directs the ionized chemical through a flow channel to an outlet which can be connected to various detectors for chemical measurements. A visual device overview can be seen in Supplemental Fig. 1.

The ZAPPI device design focuses on two primary objectives: 1) optimizing ion generation through discharge electrode design and 2) managing chemical species transport and mixing using gas microfluidic structures. Secondary design considerations include making the device easy to fixture with different ion filters, ensuring compatibility with industry-standard tube fittings (Swagelok Co., Ohio, U.S.A.) for laboratory integration, and leveraging reproducible MEMS manufacturing processes that enable medium-volume production at the wafer scale (Supplementary Figs. 2, 3).

**Fig. 1:**
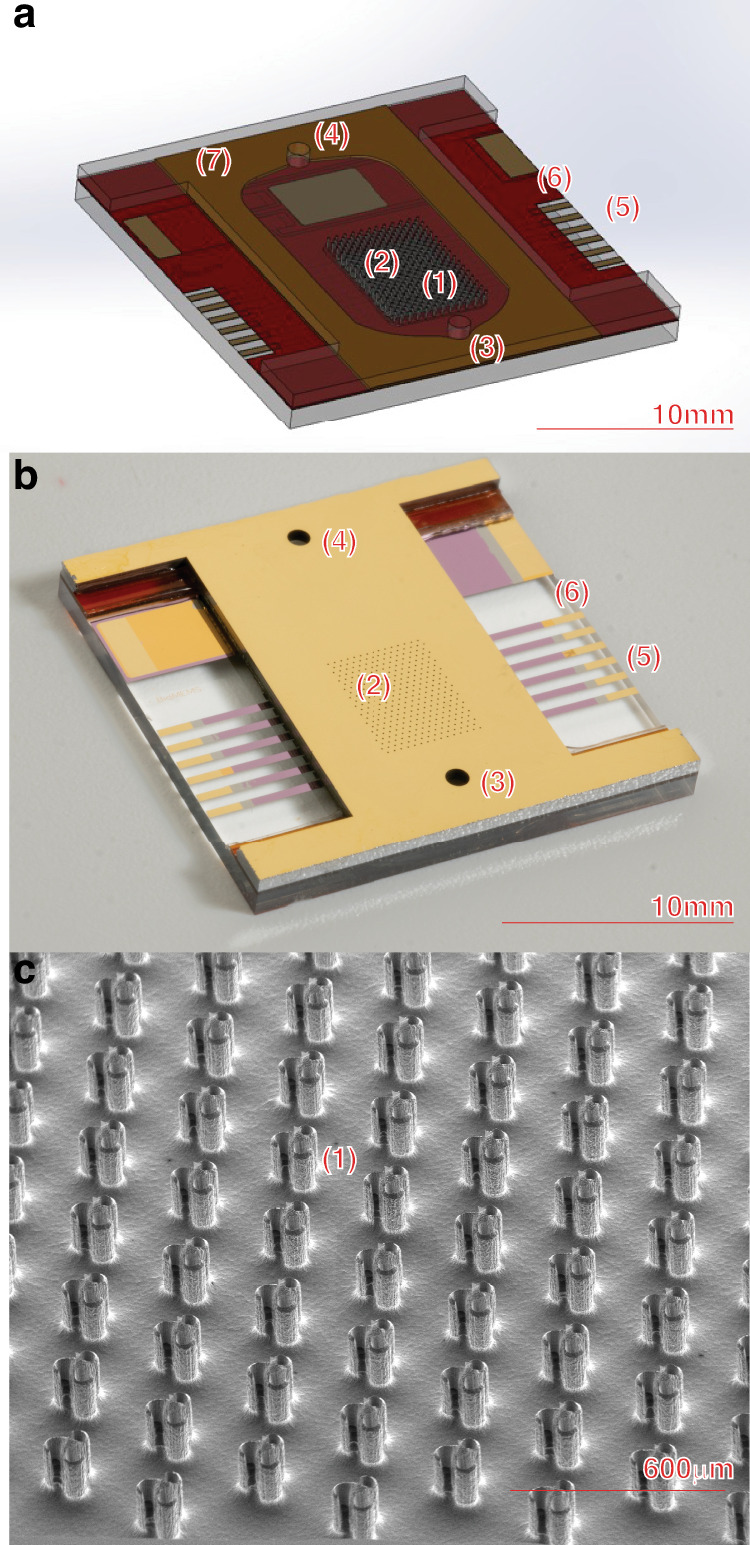
**Displays the realized device in several forms**. Features (1) depicts the hollow fluidic microneedle array plated in a thin tungsten film, (2) the analyte injector through silicon vias, (3) carrier gas inlet, (4) device outlet, (5) multiplexed tungsten cathode plates for each row of needles each with gold pads for wire bonding, (6) break-away tabs exposing electrical contacts during the dicing process, and (7) a polymer Kapton bonding layer to define the electrode gap and flow cell channel. **a** depicts a representative CAD model including the transparent (for display) silicon needle array top chip bonded to a glass substrate with an intermediate Kapton bonding layer shown in brown. The multiple layers of metal and insulated oxide are shown in gold and red, respectively. **b** displays a complete microfabricated chip. Finally, **c** shows a zoomed-in scanning electron microscope view of the silicon microneedle array

We fabricated the device using microfabrication techniques in a class 100 cleanroom (CNM2, UC Davis, CA). Its various features are depicted (Fig. 1). In operation, gases enter the chip through two distinct etched inlets. The first inlet (Fig. 1, feature 3) delivers a neutral carrier gas species such as helium or nitrogen into the flow channel. Typically, this carrier gas flows between 100 and 1000 sccm, carrying ionized species along the channel length into an ion detector. The carrier gas also serves as the working gas for the plasma discharge. The second inlet (Fig. 1, feature 2) consists of an array of 120 µm diameter holes, each leading to an individual microneedle in the device. Through these inlets, dissolved trace VOC analytes are introduced at lower flow rates, 1–15 sccm, across the entire array, for ionization and detection.

Electrically, the ZAPPI functions as a point-to-plane discharge device. The sharp-point anodes are microfabricated tungsten-plated silicon microneedles, while the planar cathodes are tungsten thin films patterned onto a glass substrate. The discharge gap is defined using a thermally bonded Kapton™ sheet (Fig. 1, feature 7) sandwiched between the silicon and glass wafers. This laser-cut Kapton also forms the bulk fluidic channel. The chip interfaces with electronics (Supplementary Figs. 4 and 5) via gold wire bonds connected to the cathode and anode electrodes (Fig. 1, feature 5).

The swirl-pattern needle shown (Fig. 1 feature 1) is conceptualized to merge fluidic and electrical discharge properties. The unique swirl pattern design delivers analytes efficiently to the ionizing discharge tip, while the sharpened geometry follows Peek’s law for the wire-cylinder model (Eq. 1), selecting parameters for positive-mode corona discharge plasma^21^

1 $${E}_{v}={m}_{v}{g}_{v}\delta ln\left(\frac{S}{r}\right)$$

Here, the critical corona voltage ${E}_{v}$ depends on the surface irregularity factor ${m}_{v}$, visual critical potential gradient ${g}_{v}$, anode radius $r$, electrode spacing $S$, and air density factor $\delta$. Mesa et al.^22^ further suggest that tip shape and geometry (denoted $k$, Eq. 2) influences the local electric field, with sharper tips producing higher field strengths at lower voltages:

2 $$E=\frac{V}{{rk}}$$

Based on these equations, we engineered the device to have sharp tips increasing localized field strength, 150 µm shanks to separate the tips from the substrate, and selected tungsten-coated silicon for mechanical durability, smooth surface finish, and enhanced secondary electron emission properties. The microneedles were fabricated using an alternating sequence of reactive ion etching (RIE) and deep reactive ion etching (DRIE), which sharpen the tip and define the shank, respectively. Complex 3D geometries, such as the swirl design, behave non-intuitively during isotropic etching. We relied on a modified computer-aided etch simulation^23^ (Supplementary Fig. 6) combined with experimental data to determine final mask dimensions. The needle structure comprises a central solid cylindrical shank with three hollow helical swirls attached. The swirls define three semi-enclosed fluidic channels running parallel to the length of the shank. Analyte is delivered to the fluidic channels by a through-silicon-via (TSV) hole (Fig. 1 feature 2) etched from the backside of the wafer to the base of the swirls, providing a direct line from the chip’s exterior to the ionizing needle tips. The helical swirls also act as anchors, suspending the needle shank over the TSV. Fully concentrically enclosed hollow needle designs, while fluidically superior, etched poorly, likely due to reactant/product transport kinetics and polymerized etch by-products in high-aspect-ratio features under low DC plasma bias. The finalized swirl design provides an acceptable channel to inject analytes while retaining manufacturability.

### Fluidic performance

**Fig. 2:**
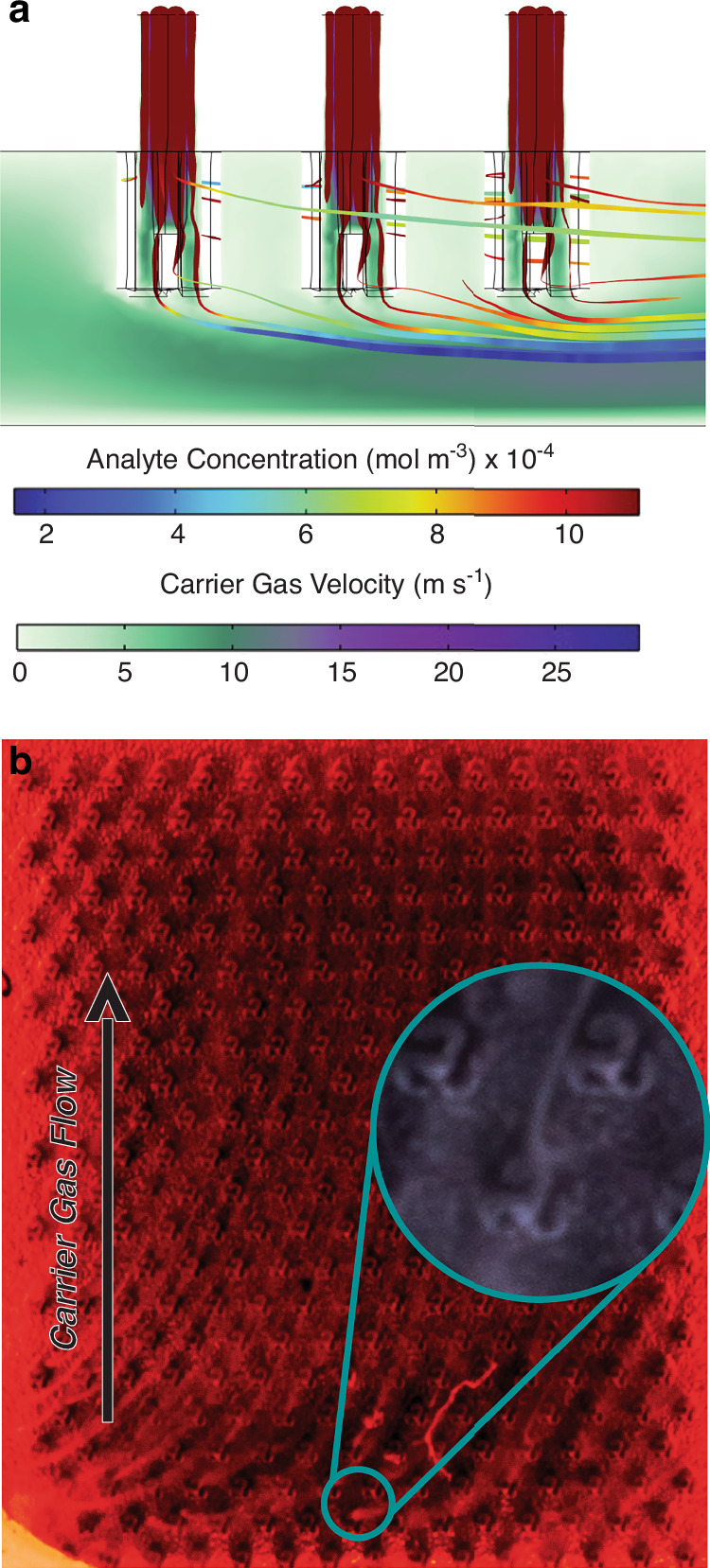
**Illustrates the microfluidic properties of the ionization chip**. **a** simulates the hollow microneedle’s analyte injection capabilities in a reduced 1-by-3 array. All parameters are scaled accordingly. An acetone inflow representing 5 sccm and 100 ppm dissolved in nitrogen is injected through the needle into the flow channel filled with nitrogen carrier gas flowing at 1000 sccm. Streamlines and arrows elucidate spatial analyte concentration as it flows through and exits the needle, while the cross-sectional slice heat map displays 2-D velocity down the midline of the channel parallel to carrier gas flow, **b** shows the injector streamlines in the actualized device wherein propylene glycol vapor is flowed through the analyte injectors into the carrier gas stream. Red LEDs side illuminate a modified clear silicone flow channel to better capture the vapor streams. A detail view shows a microneedle in white light with a vapor trail emanating from the needle tip

To further examine the microfluidics involved in transporting analytes for ionization, we present a computational fluid dynamics (CFD) model (Fig. 2a) alongside a smoke-flow visualization (Fig. 2b) of the injector structures in operation. The CFD simulation was set to a typical operating case: 1000 sccm nitrogen carrier gas and 5 sccm of 100 ppm acetone in nitrogen via the injector, with the outlet vented to atmosphere. We tracked analyte concentration shown as rainbow-shaded streamlines indicating acetone dilution, and carrier gas velocity represented by a green-to-purple gradient slice running the length of the microfluidic channel towards a detector.

Analyte injection can be seen entering the bulk carrier gas flow just past the microneedle tip. Upstream needles slow carrier gas velocity near the device top surface around the needle base, while accelerating flow along the channel’s lower surface. This velocity gradient creates a low-pressure zone just below the tips and a higher-pressure zone around the needles. In addition to the needle-analyte microfluidic interactions, we see a high-velocity carrier gas region between the needle tips and the bottom surface (purple shading, Fig. 2a). This region scavenges injected analyte, facilitating ion transport down the channel.

These simulation results were showcased experimentally in the smoke visualization. Wisps of smoke serving as a visually traceable analyte emanate from the microneedle tips and are swept down the channel by the carrier gas flow. Capturing the 3-dimensional analyte concentration field with a 2-dimensional image is accomplished by imaging at a skew angle to the chip. We used the optic’s inherently shallow focal plane to render the depth of the channel. At the bottom of the image in Fig. 2b the surface closest to the transparent cover slip is in focus. We observe the whisps of smoke traveling down the channel, consistent to the simulation data. Moving up towards the top of Fig. 2b the focal plane renders the base and shank of the microneedles. True to the simulation there is no smoke in these regions due to the higher-pressure region at the base of the needle. Qualitatively, this indicates the analyte follows the high velocity carrier gas stream between the needle tip and cover slip while remaining in low concentration near the needle base, matching the CFD results in Fig. 2a.

The visual and simulation data highlight the importance placed on delivering the analyte directly at the needle tip. In microfluidic systems, even gas systems where velocities are typically higher than their liquid-based counterparts, mixing can be challenging in laminar flow regimes^24^. With a Reynolds number Re=1480 in the carrier gas channel^25^ the system operates well within the laminar regime. Our flow simulations were verified in the device where uninterrupted continuous streams of smoke flowed from each microneedle tip to the outlet on the chip.

### Electrical performance

**Fig. 3:**
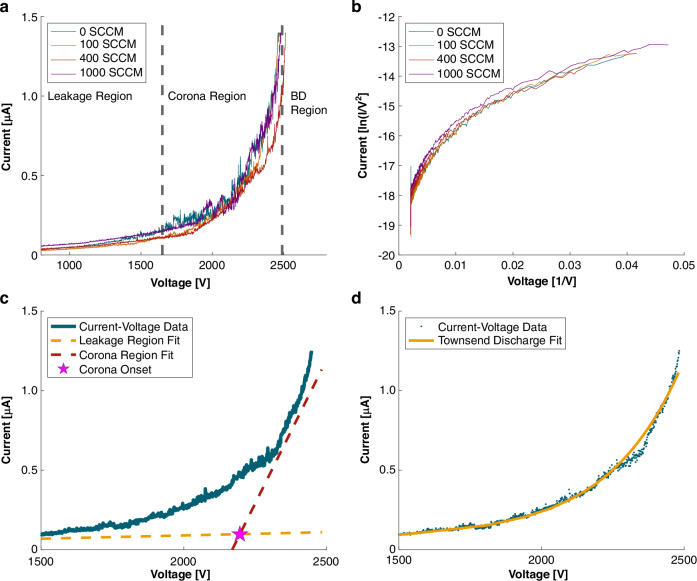
**Details device electrical properties**. **a** shows the current–voltage (*I*–*V*) relationship at various carrier gas flow rates. The linear leakage region where no charge is transferred over the electrode gap, corona discharge region, and dielectric breakdown (BD) region are each separated by the dashed lines. Similarly, at various flow rates, the I-V data is plotted to display Fowler-Nordheim electron emission in (**b**). A representative I–*V* curve at 100 sccm is plotted in (**c**) the linear leakage region in yellow and corona discharge region in red are each linearly fit, the intersection of the two linear fits at the magenta star estimates the corona onset voltage. **d** fits the Townsend discharge equation to the same 100 sccm *I*–*V* curve

Electrical discharge and field-emitting devices have been characterized by methods from high-speed photography^26^ to high-frequency current pulse analysis^27^. In ZAPPI’s development, we applied device physics to guide needle geometry and electrode gap design^28^. Here, we examine the current–voltage (I–V) relationship^21^ under controlled atmospheric conditions in enclosed submillimeter microfluidic channels. This steady-state characterization, rather than nanosecond transients, provides a practical operating model for ZAPPI’s primary function: delivering ionized chemical species to a detector (Fig. 3). Current was determined from the voltage drop across a shunt resistor at multiple carrier gas flow rates (Fig. 3a). Each dataset shows three regimes. At low voltage, the “leakage region” behaves ideally as an open circuit, though small leakage currents arise in the drive electronics under increasing voltage. In the central “discharge region,” the emitter tip’s localized high electric field ionizes gas molecules, producing a nonlinear rise in current with voltage^29^. This increased conductance was one of the first indications that ZAPPI was operating as a corona discharge device. At the highest voltages, the “breakdown region” appears when the carrier gas’s dielectric strength is exceeded, forming an arc between electrodes and triggering the 0.1 mA current trip in the power supply. We next examined the effect of carrier gas flow rate on the I–V relationship. According to Peek’s law (Eq. 1), gas density, composition, and pressure influence atmospheric-pressure discharge^29^. Across 100–1000 sccm, pressure varied by only 1.57 psi (14.64–16.21 psia). Averaged triplicate curves showed no clear correlation between flow rate and corona-region slope. This <10% pressure variation was imperceptible to the test electronics, indicating no need for pressure compensation in ZAPPI’s drive circuitry. In the same line, the pressure dependency results ruled out the necessity for analyte concentration compensation since the relatively dilute species would have insignificant contribution to the breakdown voltage. Under zero flow, however, discharge stability degraded: after ~1 min, breakdown occurred at voltages well below those in Fig. 3a, likely due to ion accumulation in the confined channel suppressing breakdown voltage.

Alluded to previously, corona discharge is the primary mechanism for generating the atmospheric pressure plasma. To investigate further, we plotted the current voltage data in the Fowler-Nordheim (FN) form for field emitters^30^. For the ideal field emitter, the relationship between current density and the inverse of the electric field should be linear indicating the work function of the emitter material. Figure 3b clearly shows nonlinearity indicating several deviations from the ideal field emitter, geometric effects caused by the sharp needle points, multiple discharge sites, and most importantly the presence of other electrical discharge effects^31,32^. The needle-to-plane geometry and atmospheric operation are consistent with corona discharge, confirmed by the observed purple corona glow at the tips (Supplementary Fig. 7).

The onset voltage, the minimum for a self-sustaining avalanche discharge defines the practical lower bound for operation, and the breakdown voltage the upper bound. Using the technique described by Huang et al.^33^, we found the intersection between linear fits for the leakage and discharge regions (Fig. 3c). For the ZAPPI device, we found the onset voltage to be 2170 ± 77 V, 2188 ± 23 V, 2199 ± 8 V, 2177 ± 25 V, at 0 sccm, 100 sccm, 400 sccm and 1000 sccm carrier gas flow rates, respectively. Our specific electronic configuration uses a ballast resistor to enhance stability by limiting current, meaning this estimate will overreport the device onset voltage due to the added resistance in the system.

To further complete the electrical testing parameters, we fit an exponential curve (Fig. 3d) to the current-voltage data directly from Townsend discharge relationships with Henson’s^34^ point-to-plane geometry approximations in Eq. 3. Where *V*_0_ describes the onset voltage and A represents a physical parameters coefficient.

3 $$I={AV}\left(V-{V}_{0}\right)$$

Meng et al^35^. in their more recent findings describe the effect of electrode geometries like the ones fabricated for ZAPPI. An exponent, *n* typically ranging from 1.5 to 2.0 describes the *I*–*V* relationship more precisely accounting for differing electrode geometries and interelectrode distances. A compensatory factor K replaces A in Eq. 4 to account for various ambient conditions like relative humidity, temperature, and pressure.

4 $$I=K{\left(V-{V}_{0}\right)}^{n}$$

After fitting the data, we determined the exponent value *n* to be 1.9 ± 0.17. In Meng’s review of other point-to-plane corona discharge devices with their revised fit method, they found electrode configurations with small electrode point radii—sub 15 µm radius of curvature and low inter-electrode distances—sub 30 mm^34,36^ yielded an exponential fit *n* close to 2.0. The ZAPPI’s isotropically etched microfabricated needle structure results in sharp needle tips, pushing the device’s exponential fit term close to the ideal condition *n* = 2.0.

### Chemical test performance

Chemical testing was performed using two ion-separating detectors. First, we coupled ZAPPI to an Orbitrap mass spectrometer, the gold standard for identifying chemical compounds by molecular weight. Single-component volatile organic compounds (VOCs) were injected into the ZAPPI chip to verify the formation of ionized species. As a proof of concept for portable applications, we also integrated ZAPPI with a custom-built portable Faraday cup-style ion current detector to demonstrate future device integrations.

**Fig. 4:**
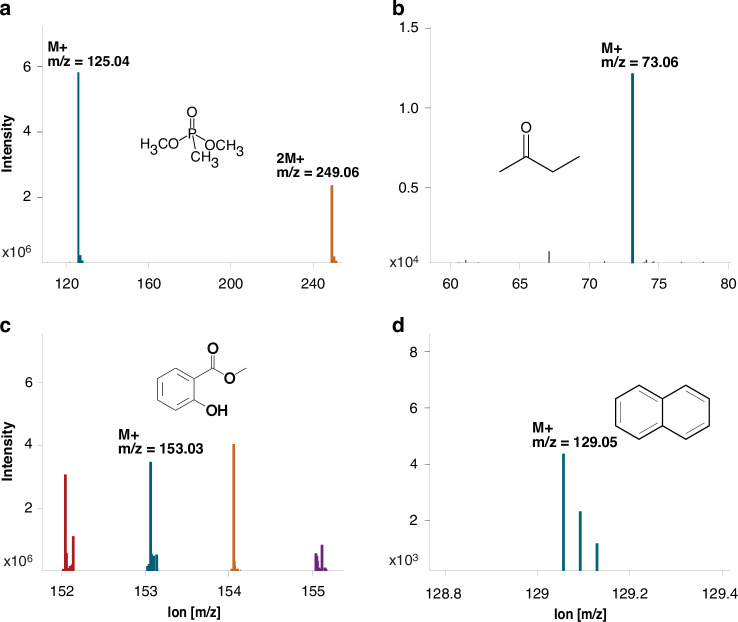
**Mass spectra derived from various chemicals into the atmospheric pressure inlet of an orbitrap mass spectrometer ionized by the device.** **a** Monomer [M+] and dimer [2M+] peaks from DMMP, a common chemical warfare precursor is shown over a wide m/z range to highlight the relative signal intensity and the coexistence of monomer and dimer ion species in the spectra. **b** 2-butanone monomer [M+] a metabolomic biomarker used in health monitoring, **c** Methyl salicylate [M+] the wintergreen flavoring agent, and **d** Naphthalene monomer [M+] a common environmental toxicant are targeted and identified operating the mass spectrometer in single ion mode to more efficiently target the peaks associated with a strong peak characteristic of each chemical species

Figure 4 presents representative mass spectra for several VOCs. Device functionality was validated by introducing single-component VOCs of known mass into the ZAPPI chip. With the mass spectrometer’s default ionization source disabled, the detection of protonated monomer peaks at the expected masses for each target analyte provides strong evidence that the ZAPPI chip generates ionized VOC species. In Fig. 4a, injection of 4.13 ppm dimethyl methylphosphate (DMMP), a common simulant for the nerve agent Sarin, produced two dominant peaks at m/z = 125, corresponding to the protonated monomer ion, and at m/z = 249, reflecting a protonated dimer. Although this study focuses on monomer ions for confirmatory analysis, the appearance of dimer ions highlights ZAPPI’s ability to generate higher-order species for improved chemical fingerprinting.

We next challenged the device with less easily ionized compounds: 2-butanone, methyl salicylate, and naphthalene (Fig. 4b–d). These species are less stable than DMMP but were all successfully detected by operating the mass spectrometer in single-ion monitoring (SIM) mode at the expected monomer m/z. The selected compounds represent diverse analyte classes relevant to portable detection needs.

**Fig. 5:**
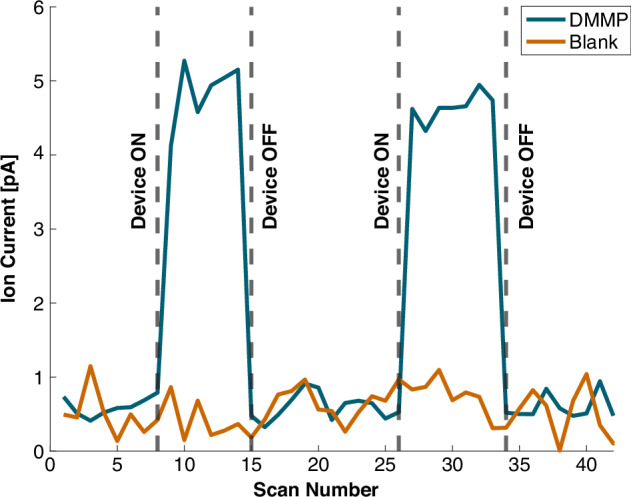
**The ZAPPI ionization source coupled to a faraday plate stye ion counter.** A 250 ppm DMMP sample is injected into the ZAPPI chip via a 10 sccm analyte flow rate resulting in $\dot{m}=23\,{\mathrm{ngs}}^{-1}$ and a 1000 sccm carrier gas flow rate. The blue trace follows the detector current as the ZAPPI source is switched on and off ionizing the sample VOC. The orange trace follows the ion current with no additional VOC added and just the carrier gas plasma

ZAPPI deviates from Spindt’s seminal paper on microneedle array emitters for chemical ionization in that it operates at atmospheric pressure. Coupling to a mass spectrometer like the orbitrap in Fig. 4 demonstrates chemical species identification with an atmospheric pressure detector, but the full value of the ZAPPI source comes is when it is coupled with complimentary portable detectors. Ion current generated by ZAPPI measured by a Faraday plate is plotted in Fig. 5. The source is switched on and off to compare the baseline signal versus the ion current with the chemical sample. We detected the 250 ppm DMMP delivered at a mass flow rate $\dot{m}=23\,{\mathrm{ngs}}^{-1}$ with 10 sccm analyte gas flow rate and 1000 sccm carrier gas flow rate as a change in detector signal averaging 4.74 ± 0.32 pA, a factor of 8 above the 0.59 ± 0.16 pA noise floor. We use the “blank” run with no added VOC to confirm the increase in ion current is a result of ionized DMMP and not electrical noise generated by the plasma. This result alludes to a promising concept where ZAPPI could be integrated with a more powerful ion filter for portable chemical species identification.

## Conclusions

In this work, we present the design, fabrication, and evaluation of the ZAPPI chip, a MEMS plasma ionization source engineered as a component for highly selective and sensitive chemical sensing. We demonstrate the use of a high-aspect-ratio hollow microneedle structure incorporating microfluidic and electrical discharge properties to achieve localized, stable corona discharge at atmospheric pressure.

The swirled-pattern microneedle geometry designed to deliver analyte directly to the high energy plasma region was optimized using computational fluid dynamics and verified with experimental visualization. Carrier gas and analyte gas velocity profiles and analyte concentration profiles were investigated then demonstrated to confirm the analyte focusing abilities of the device at the discharge needle tip.

Electrical testing confirmed the corona discharge conditions across a range of carrier gas flow rates, and therefore pressure conditions. Characteristic I-V curves confirmed the clear transition between the linear leakage current region, the exponential electrical discharging corona region, and breakdown regions. Fowler-Nordheim analysis indicates the deviations from an ideal field emitter, reinforcing the idea that an avalanche corona discharge is the likely discharge mechanism. For practical applications of the device, we use two curve fitting methods, a linear intersection method, and exponential fit to calculate onset voltage, corona discharge exponent n, and compensation factor K.

The most critical performance benchmark, chemical detection performance was validated through mass spectrometry and by coupling to a custom-built electrometer ion detector. ZAPPI consistently ionized compounds and was able to detect analyte at trace levels of $\dot{m}=23\,{\mathrm{ngs}}^{-1}$ mass flow rate DMMP when coupled with the custom detector. The detected compounds from several compound families represent point-of-sampling/point-of-need targets. DMMP, a simulated nerve agent, was detected in monomer and dimer ion forms. 2-butanone is a commonly studied biomarker in exhaled breath metabolomics, with elevated levels linked to smoking-related lung damage and lung cancer^37^. Methyl salicylate (wintergreen) is widely used as a flavoring agent but has also been investigated as a low-impact attractant for spotted lanternfly pest management^38^. Naphthalene belongs to the polycyclic aromatic hydrocarbon (PAH) family, a class of combustion by-products with well-documented toxicological importance. Naphthalene is present in wildfire smoke and classified as carcinogenic, mutagenic, and toxic^39^. By enabling its detection, ZAPPI could form the basis of a next-generation, real-time personal exposure monitor for firefighters and other at-risk populations.

ZAPPI distinguishes itself from prior work by offering an arrayed silicon microneedle device relying on common state-of-the-art etching, thin-film deposition, and packaging microfabrication processes. We also demonstrate as few others have before a miniature atmospheric pressure plasma discharge ionization source validated for chemical analysis. To our knowledge, ZAPPI has shown the lowest limit of detection. We anticipate several future studies stemming from this proof-of-concept. Foremost, ZAPPI will be integrated into a portable DMS platform and ultimately, we envision ZAPPI as a cornerstone component to a monolithically fabricated portable trace gas analysis platform on a chip.

## Materials and methods

### Silicon microneedles

The microdischarge chip is fabricated in the Center for Nano-Micromanucaturing, a class 100 clean room on the UC Davis campus. A step-by-step, detailed schematic overview of the process can be seen (Supplemental Table 1). The microneedle electrode device uses a 150 mm × 675 µm thick <100> p-type silicon substrate. On the top side, the microneedle device is patterned in a 20 µm layer of Futurrex NR78-8000p negative photoresist and hard-baked at 150 *°*C overnight in vacuum conditions. An isotropic inductively coupled reactive ion etch (ICP-RIE) (Plasmatherm, Apex, Supplementary Fig. 8) process is performed to define the tapered needle point, undercutting the mask laterally while the etch proceeds downward. A combination of SF_6_/O_2_/CHF_3_ process gasses at 80/5/20 SCCM flowrates respectively in 100 mtorr chamber pressure are ignited with 1Kw ICP plasma and 80 W DC platen bias to achieve the isotropic etch characteristics. The SF_6 plasma_ accomplishes the bulk silicon etching while O_2_ and CHF_3_ remove polymerized silicon oxyfluorides. Removing polymerized byproducts chemically in the absence of a higher DC bias is essential to consistent and speedy bulk material removal. Next, the wafer is deep-silicon etched (Plasmatherm, Versaline, Supplementary Fig. 9) using the BOSCH process to define the high-aspect-ratio sidewalls. Needles measuring 120 µm in diameter and 280 µm pitch are etched anisotropically to a nominal depth of 150 µm. The deep silicon etching process further differentiates the needle geometry from the substrate to ensure the electrical discharge is localized to the needle tips during device operation. Additionally, the needle height provides allowance for the backside TSV etch, preventing needle over-etching. Completing the top side etching, the same isotropic RIE-ICP etch recipe is used to undercut etch and sharpen the needle points. A diagram of this process can be seen (Supplementary Fig. 10). Finally, a 250 nm sputtered (Kurt J. Lesker, Labline) tungsten layer with chromium adhesion is deposited onto the microneedles, while the surrounding features are covered in a 500 nm silicon dioxide film deposited with plasma enhanced chemical vapor deposition (PECVD) (Plasmatherm Vision310) for insulation.

The silicon device wafer is then flipped over for backside etching, patterning the through-silicon via gas ports with the same 20 µm thick NR78-8000P resist. The device wafer is temporarily bonded to a sacrificial carrier wafer using silicone thermal grease (SantoVac, 5 PPE), preventing helium chuck coolant leakage during the through etch process. The wafer is then etched through using the Bosch deep silicon etching process. Between the 1 mm diameter carrier gas ports, 100 µm diameter analyte injects, and electrical contact windows (Fig. 1, features 2, 3, 4, 6), each feature has a different aperture size and therefore different etch rate due to the etch loading and product removal kinetics. The previously mentioned needle height allows for each feature to be etched through without over etching and destroying the needle tips during this process. Finally, electron beam evaporation (e-beam) (CHA, E-beam evaporator) is used to deposit a 200 nm gold film, providing a wire bonding surface connected to the needle array.

### High-voltage glass cathode wafer

The cathode electronics are patterned on a separate 150 mm × 1000 µm thick Borofloat33 wafer using 1.8 µm spin-on NR9 photoresist for each layer. A 250 nm sputtered tungsten film is first deposited for lift-off to define the cathode electrodes. Next, a 250 nm PECVD silicon dioxide layer insulator. Next, a 200 nm e-beam gold film is deposited to form the electrical wire bonding pads. Finally, a second silicon dioxide PECVD layer is deposited to improve insulation and protect the electrodes from delaminating.

### Bonding and packaging

Both the silicon microneedle device wafer and Borofloat33 cathode wafer are bonded together with an intermediate heat-bondable, chemically inert 5 mil thick Kapton 18-FN-1F2 film stacked two layers approximately 250 µm thick after compression bonding. A sheet is laser-cut to define the 7.5 mm-wide fluidic channel of the device. In a wafer bonder (EVG, 501 wafer bonder), the silicon wafer is placed needle side up atop the bonding fixture. The two Kapton sheets are placed onto the silicon wafer using 1 mm dowel pins temporarily inserted into the inlet and outlet ports in the wafer to align the film sheets. The bonding fixture is inserted into a mask and bond aligner (EVG, 620 mask aligner, Supplementary Fig. 11), where the Borofloat33 cathode wafer and silicon-Kapton stack are aligned using fiducial alignment marks on both wafers. The silicon-kapton-Borofloat33 stack is thermally pressed together at 1 kN and 250 °C for 5 min.

Wafers are diced into 20 mm × 20 mm dies. During the dicing process, etched features on the chip (Fig. 1 feature 6) are released, revealing the gold wire bonding pads on the cathode wafer. Individual chips are glued to a breakout printed circuit board (PCB) seen with cyanoacrylate before gold wire bond connections are made between the two (Supplemental Fig. 4). To complete the device a CNC machined polyether ether ketone (PEEK) fixture (Supplementary Figs. 12, 13) is fastened to the PCB, protecting the chip, and providing threaded holes to interface 1/16” tubing to the microfluidic structures.

### Microfluidics analysis

Fluidic simulations were computed on COMSOL 6.2 using the laminar flow and transport of diluted species physics modules. Simulations were designed around the typical operating conditions of the device. The simulation was simplified by taking a 3 by 1 cross-section of the needle array to minimize computational time while still capturing the complexities of upstream and downstream needles on the injection flow dynamics. Inlet conditions are scaled accordingly to represent a 1 slpm carrier gas flow rate, 5 sccm analyte flow rate, and 100 ppm acetone analyte. The normal mesh size, GMRES (fluidics), PARDISO (diluted species) solvers were used. In addition to the microfluidic analysis performed on the needles, (Supplemental Figure 14**)** shows additional simulation data used to engineer the flow channel.

The device was photographed using a 60 mm 2:1 macro lens attached to a Sony mirrorless digital camera. For the photograph a special electrically non-functional version of the chip was fabricated using a transparent Borofloat33 glass without cathode electrodes and a 1/16” thick translucent silicone gasket replacing the Kapton film. These modifications were necessary to capture the microneedles, fluidic channel, and appropriately light the photograph. Fiberoptic strands illuminated by a 10 W multicolor LED light source were inserted into the fixture, butting the terminated end against the silicone gasket to best side-illuminate the channel. A photograph of this apparatus can be seen (Supplementary Fig. 15). A polyethylene glycol vapor, referred to as “smoke”, was generated with a visual effects smoke machine (Ulanzi, FM01) and dispensed into a Tedlar 3 L Gas Sampling bag (Sigma Aldrich, St. Louis, MO). A nitrogen carrier gas set to 100 sccm was supplied to the inlet port, and the smoke was squeezed from the bag into the microneedle injector port inlet. Photos were captured with an aperture of f.22 at 1/250 s exposure.

### Electrical analysis

The previously mentioned circuit board described was designed to power the chip and measure current-voltage properties (Supplemental Fig. 5). The PCB includes high voltage terminals connected to an external power supply, a diode to prevent back current, a current restricting 10 MΩ ballast resistor, a 100 kΩ shunt resistor to measure device current, and terminals for attaching test equipment. Gas is supplied to the test apparatus with a mass flow controller (Apex Vacuum LLC, Woodstock, GA) connected to a high-purity nitrogen gas cylinder (Airgas NI-UHP300). A high-voltage power supply (Sanford) supplies voltage to the device, resulting in the high electric field between the needle anode (+V) electrodes and the cathode plate (ground) patterned onto the glass wafer. The function generator producing a ramp signal from 0 V to 10 V over a 240 second time window is connected to the HV_SET terminal on the high-voltage supply. Over the 0–5 kV DC range available on the voltage supply, the configuration resulted in a 20.83 V s^−1^ ramp delivered to the chip. This rate was chosen to minimize voltage tracking error between setpoint and delivered voltage. While the voltage increases, a multimeter (Keysight, 3458 A) measures the voltage across the shunt resistor to calculate current. Using the function generator’s trigger signal, the calculated current from the multimeter can be synced to the calculated voltage output from the high voltage power supply. A photograph of the apparatus can be seen (Supplementary Fig. 16).

### Chemical sensing analysis

Mass spectra were obtained using a 2020 Thermo Q-Exactive High-Field Orbitrap mass spectrometer, analyzed with the OpenChrom software package. The mass spectrometer was modified by removing the electrospray ionization (ESI) source from the MS atmospheric inlet and fitting a custom fixture for the ZAPPI source, placing the outlet of the chip 3 mm from the entrance (Supplementary Fig. 17). Without tubing, ZAPPI sprays ions directly at the focusing inlet cone. Using two MFCs attached to the ZAPPI device, the nitrogen carrier gas flow rate was set to 500 sccm and analyte injection flow was set to 15 sccm. A Swagelock tee fitting with one end terminated in a septum was placed between the analyte injection MFC and chip. During analysis, analytes are injected through the septum with a syringe, introducing the chemicals into the ionization source. The ZAPPI chip was powered without the ballast resistor using an 850 V current-controlled PWM power supply to prevent harmful dielectric breakdown. For DMMP, data were collected from 50 to 350 *m/z*. The remaining chemicals were measured in single-ion mode detection, set to scan a 50 Dalton wide window with the primary monomer ion centered. Each compound was injected into the device, analyzed, and allowed to clear under a nitrogen purge for approximately 10 min before the next chemical was injected.

Portable device testing was performed in a similar manner to the mass spectrometry testing. Instead of using the custom rail fixture to position the chip in front of the atmospheric inlet, an adapter plate was made to interface the ZAPPI to a custom-made Faraday plate ion counter. The detector chip comprised of two parallel ceramic plates sealed with an EPTF gasket with patterned gold planar ion collectors. These are fixtured in a machined aluminum housing that resulted in a ZAPPI to detector length of 13.4 mm. Electrical current was read from the Faraday plate using an electrometer (GAA Custom Electronics, LLC). In this configuration, several modifications were made to the ZAPPI chip accommode the different detector. The electrode gap was increased to 500 µm matching the parallel plate electrometer gap spacing, and the carrier gas flow was changed to 1000 sccm helium suppressing the onset voltage to accommodate the doubled interelectrode gap. First, a blank is measured with the device on and only carrier gas plasma. Then, a 250 ppm DMMP sample was diluted in nitrogen in a 3 L Tedlar bag. A syringe was drawn and injected into a septum in-line with the 10 sccm helium analyte flow resulting in 23 ng/s DMMP delivery rate. An experimental apparatus can be seen (Supplementary Fig. 18).

## Supplementary information

Supplemental Material
