# Supplementary material for: A silicon microneedle array atmospheric pressure plasma ionization source for real-time trace gas chemical analysis
Source: Microsyst Nanoeng. 2026 May 21;12:197. doi: 10.1038/s41378-026-01291-4 (PMC13194937; doi:10.1038/s41378-026-01291-4)
Supplement: Supplementary file 1 — Supplemental Material [file 41378_2026_1291_MOESM1_ESM.docx]

Supplemental Material

# A Silicon Microneedle Array Atmospheric Pressure Plasma Ionization Source for Real-Time Trace Gas Chemical Analysis

Bradley Chew^1,3^, Dylan T. Koch^2,3^, Patrick Gibson^1,3^, Mitchell M. McCartney^1,3^, Eva Borras^1,3^, Nicholas J. Kenyon^3,4^, Cristina E. Davis^1,3,*^

^1^ Department of Mechanical and Aerospace Engineering, One Shields Avenue, University of California, Davis, Davis, CA

^2^ Department of Electrical and Computer Engineering, One Shields Avenue, University of California, Davis, Davis, CA

^3^ UC Davis Lung Center, Davis, CA

^4^ Department of Internal Medicine, UC Davis, Sacramento, CA

*Correspondence [cedavis@ucdavis.edu](mailto:cedavis@ucdavis.edu)

Device Overview


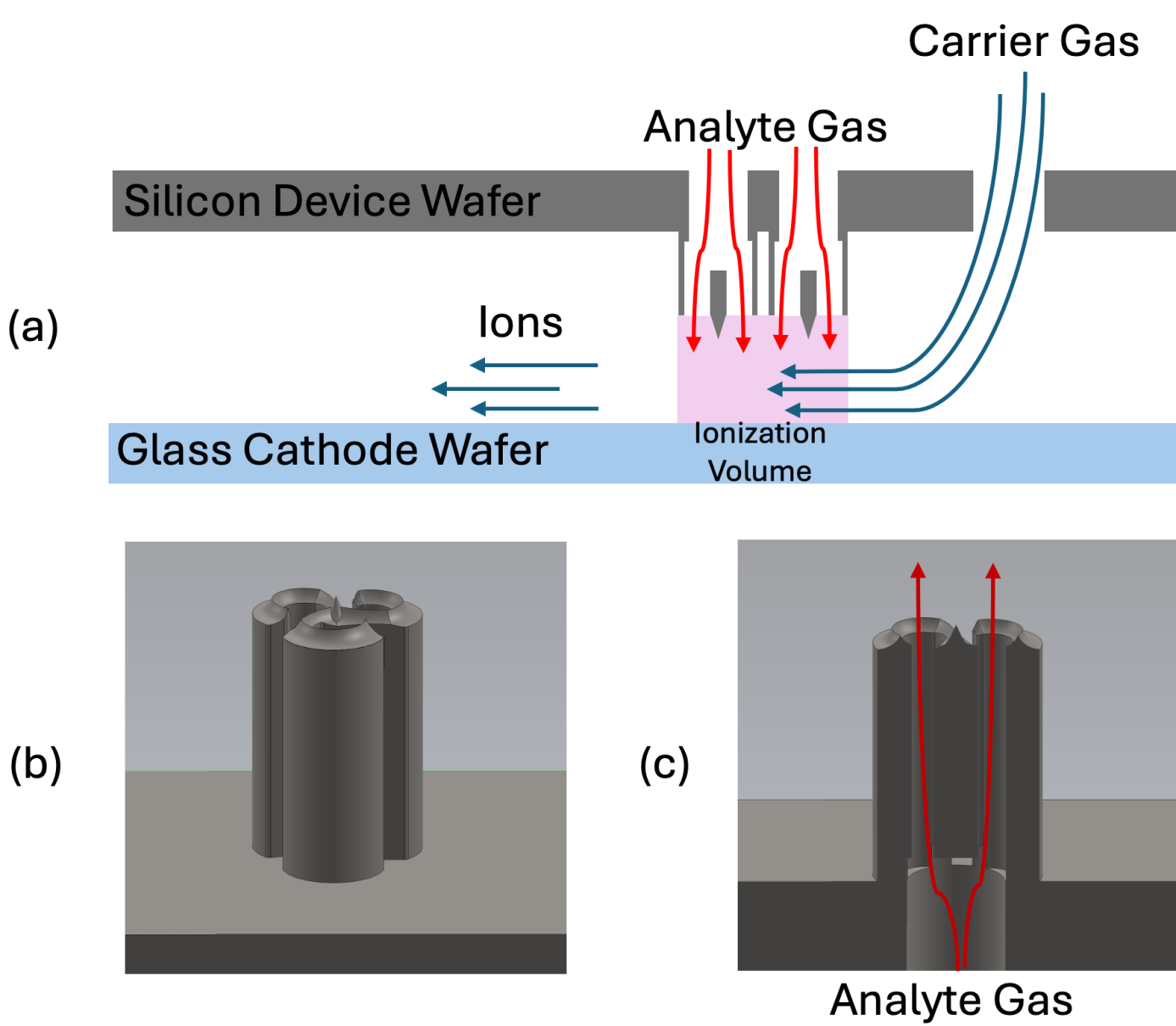


Supplemental Figure 1: A stylized schematic overview of the operational principals of ZAPPI. (a) shows ZAPPI in a cross section view, carrier gas enters through a large via (also seen as Figure 1, feature 3), meanwhile analyte is injected through the hollow needles (also seen as Figure 1, Feature 1,2), the analyte is ionized by the plasma at the needle tips, and a resultant stream of ions in carrier gas is directed downstream towards a detector. (b) shows a 3D rendered needle in a side view, and (c) the same needle side view but in a cross section. (c) demonstrates how the outer hub constrains analyte gas towards the suspended needle tip.

Wafer Production Photographs


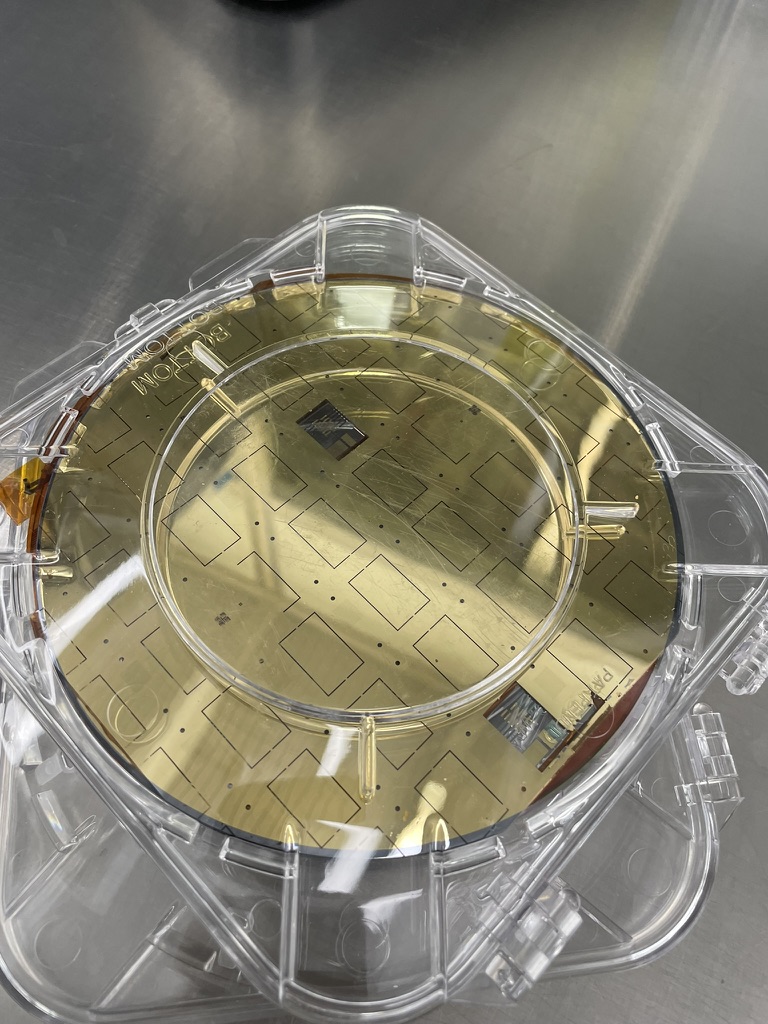


Supplemental Figure 2: A topside photograph of a completed ZAPPI wafer. Notably in some locations the wing-like structures have already release from the wafer before dicing revealing the wire bond pads on the BF-33 wafer below.


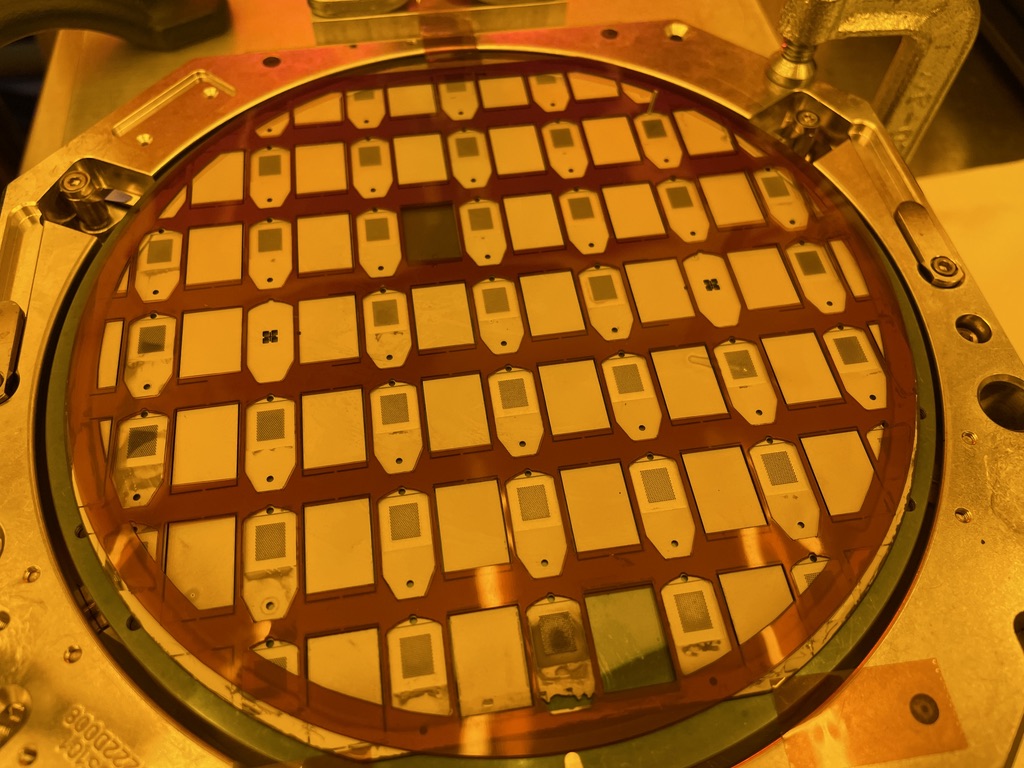


Supplemental Figure 3: A completed pre-diced ZAPPI wafer immediately after bonding. This wafer has transparent ITO electrodes instead of the typical Tungsten/Gold electrodes to show the microneedles more clearly. The orange-brown Kapton bonding layer can also be seen clearly defining the flow channels.

Test Electronics

Supplemental Figure 4: Layout design for the ZAPPI interface board.

Supplemental Figure 5: Schematic for the ZAPPI interface board. The components labeled HV_JMP are air gapped 0Ω resistors used for activating individual rows on the array. 14

Needle Etch Simulation


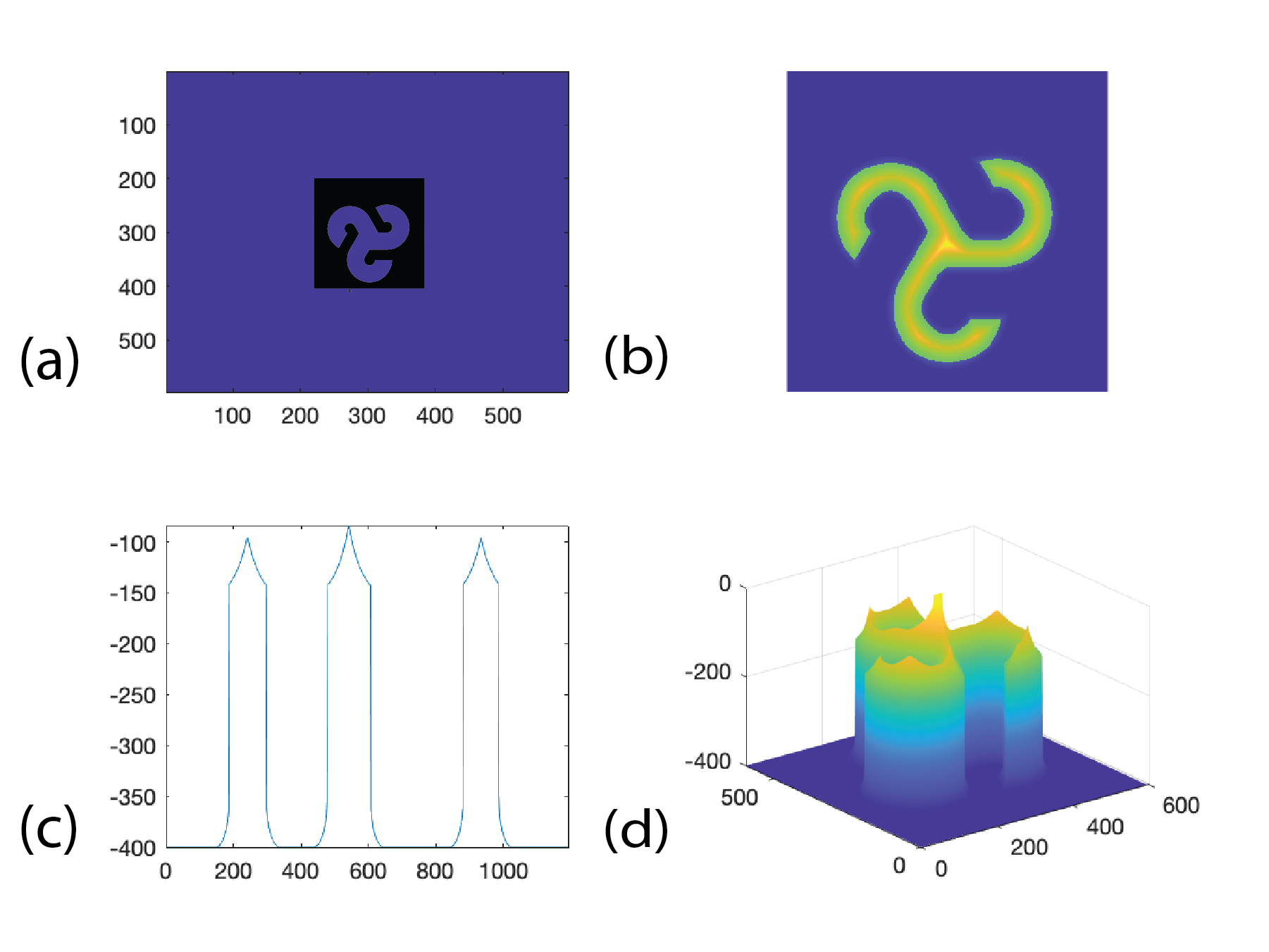


Supplemental Figure 6: Isotropic etching simulation for Sulfer Hexaflouride SF_6_ . The simulation takes a mask input, etching depth parameters and outputs a 3D geometry, and structure profile. The simulation accounts for the crystallographic plane isotropy factor. (a) shows the 2D mask profile, (b) a top down post etch view, (c) a center slice cross-section view of the etch, (c) 3D isometric view of the etcher needle.

Corona Discharge Photograph


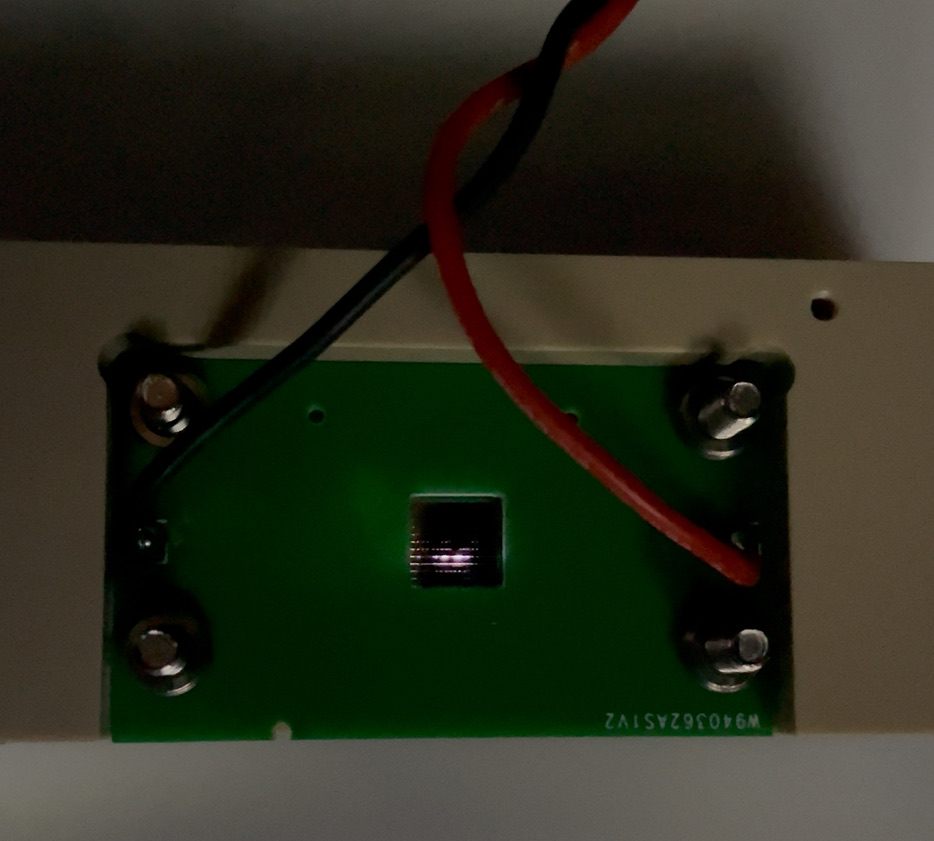


Supplemental Figure 7: The ZAPPI chip with no carrier gas flow being powered for the first time. There is a purple plasma glow along the row of cathodes connected to ground.

Process Parameters and Description

Supplemental Figure 8: The isotropic ICP-RIE recipe. The 12 cycles shown represent the post DSE etch, but the same recipe is used for both isotropic etching steps.

Supplemental Figure 9: Deep silicon etching recipe powered by 3kw ICP plasma.


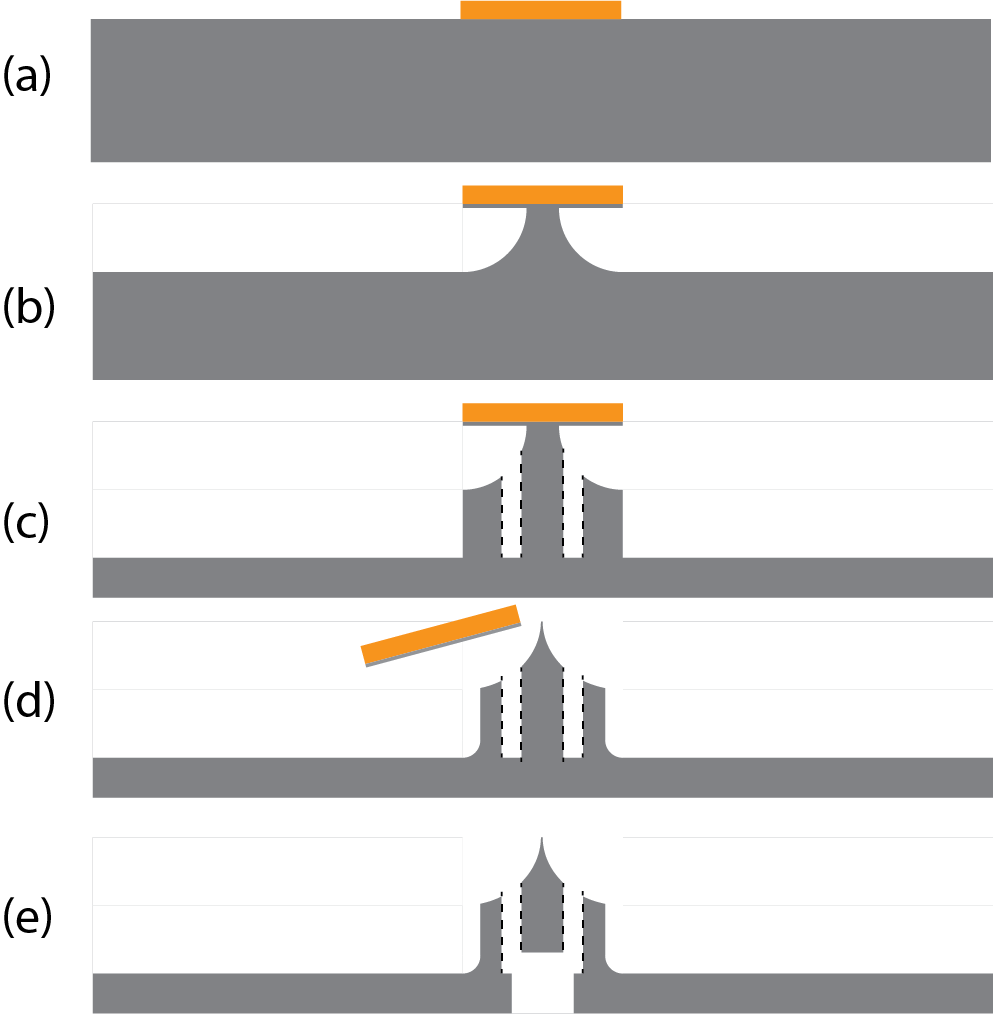


Supplemental Figure 10: An abridged etching overview for the microneedle designs showing additional detail on the needle tip formation mechanism in a cross-section view. Dashed lines indicate segments where the needle is supported by the spoke-like stricture. (a)A photoresist layer is patterned and hardbaked), (b) the first ICP-RIE isotropic etch defines the needle tip while leaving some contact with the photoresist mask above, (c) deep silicon etching is used anisotropically to define the needle shank, (d) a second ICP-RIE etches the remaining silicon attaching the resist hat to the needle until the hat snaps off leaving a sharp tip, (e) the backside DSE hollows out the needle and reveals gas vias.

Supplemental Figure 11: Bonding recipe for the Kapton thermal compression.

Supplemental Table 1: A complete fabrication plan for the ZAPPI chip.

Engineering Drawings

Supplemental Figure 12: The PEEK test fixtures used for electrical and fluidic testing. There are #10-32 tapped holes to accommodate tube fittings, screw holes to fasten the fixture to the circuit board, and a pocket used to gasket seal against the chip’s through silicon vias.

Supplemental Figure 13: A second version of the fixture was made to hold the chip where one of the ends is open. In this configurations, ions flow straight out of the end of the ZAPPI chip and into the mass spectrometer.

Fluidic Channel Simulation


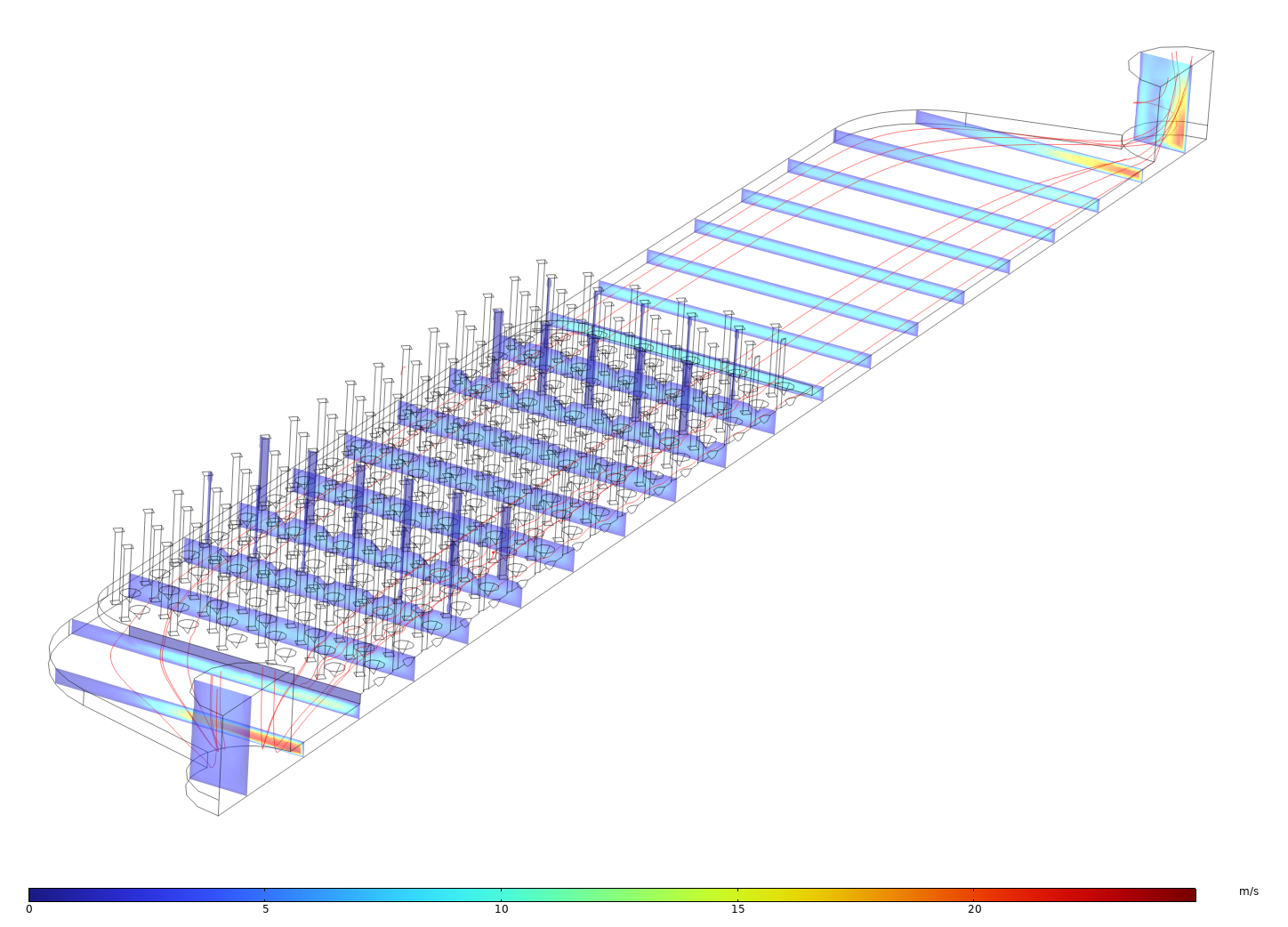


Supplemental Figure 14: A velocity field COMSOL simulation of the ZAPPI chip displayed with a slice plot. Red streamlines display the direction of carrier gas flow through the microchannel.

Experimental Apparatuses


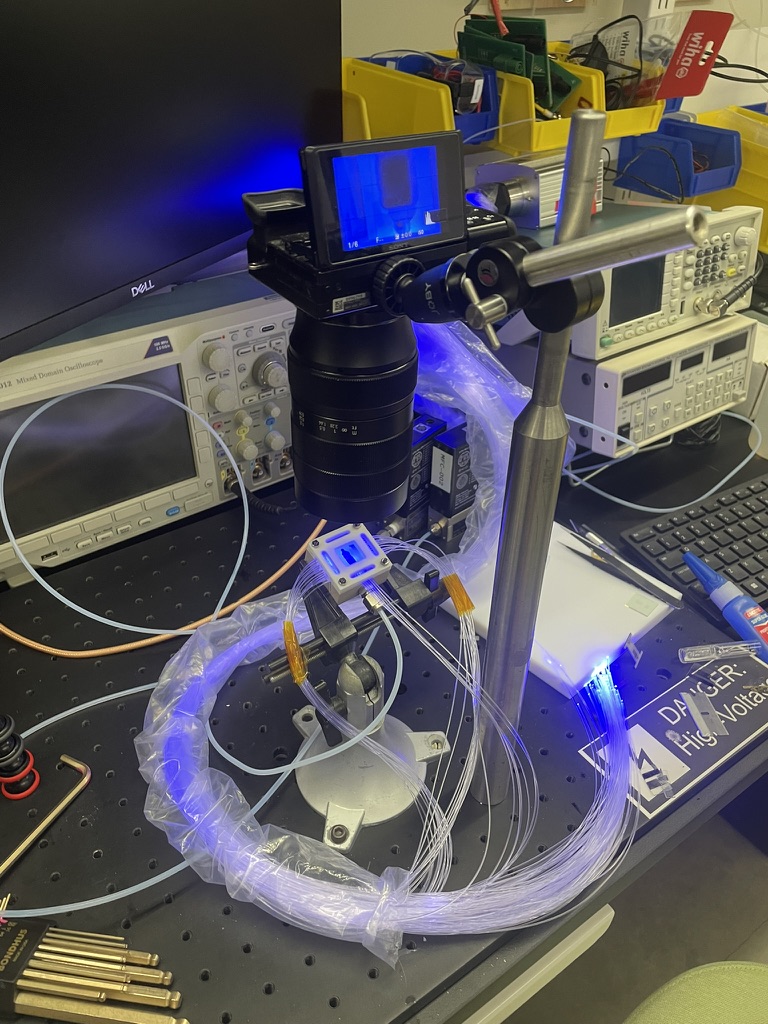


Supplemental Figure 15: Photographing apparatus for the smoke test. There are fiber optics illuminated by a multicolor LED side illuminating the ZAPPI chip. A macro lens and mirrorless camera are used to capture the image. Various fluidic connections flow gas and smoke through the chip to emulate analyte flowing through the chip with smoke.


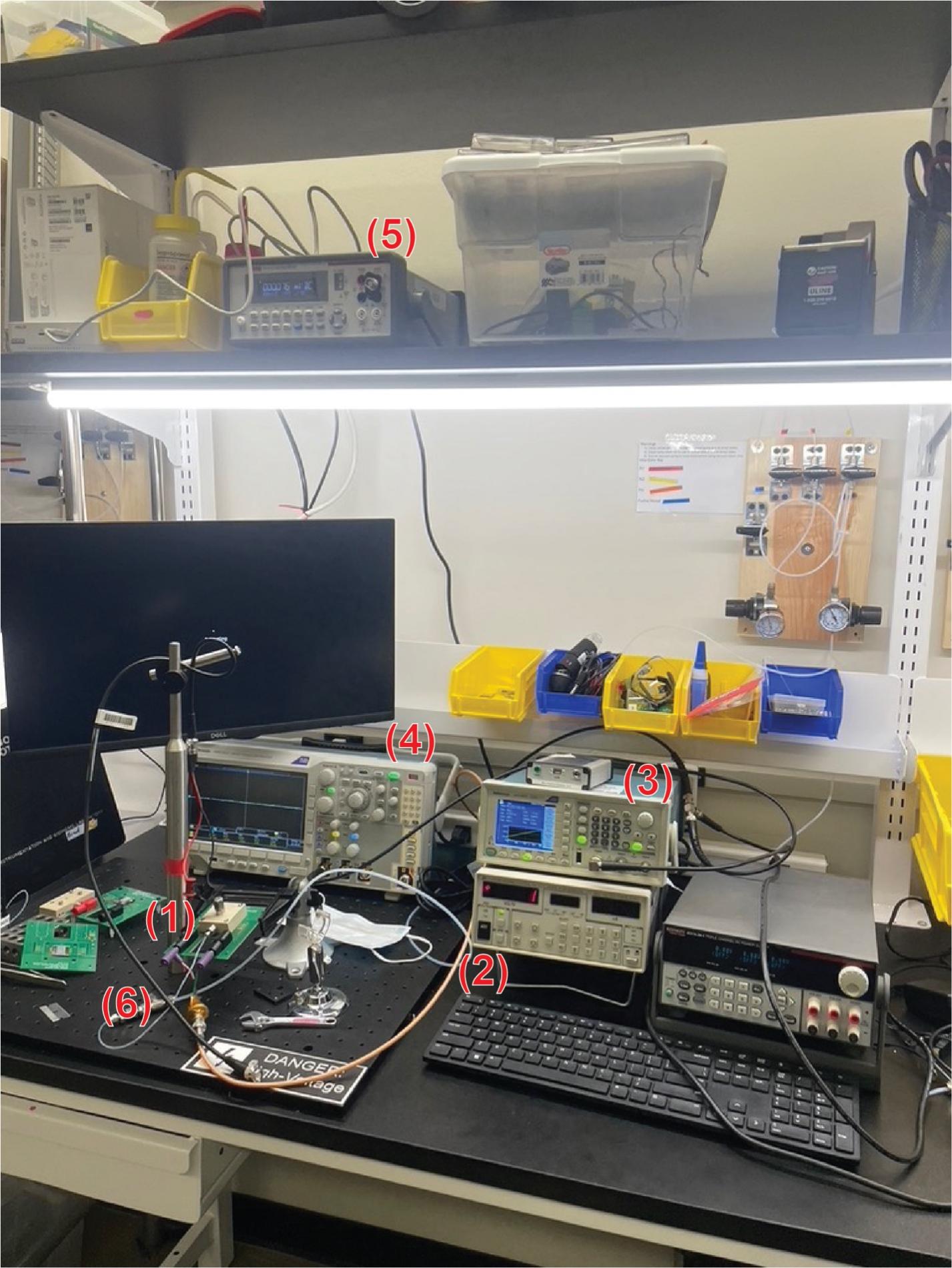


Supplemental Figure 16: The experimental setup used to generate the current voltage data. A function generator is monitored with an oscilloscope and outputs a signal to ramp the high voltage power supply. A high precision, high voltage digital multimeter is used to measure the voltage across a shunt resistor to calculate the corona discharge current. (1) ZAPPI fixture and electrical test specific PCB, (2) high voltage power supply, (3) function generator for dynamically controlling the high voltage power supply, (4) oscilloscope to measure control signal, (5) precision multimeter monitoring corona current, (6) carrier gas supply line.


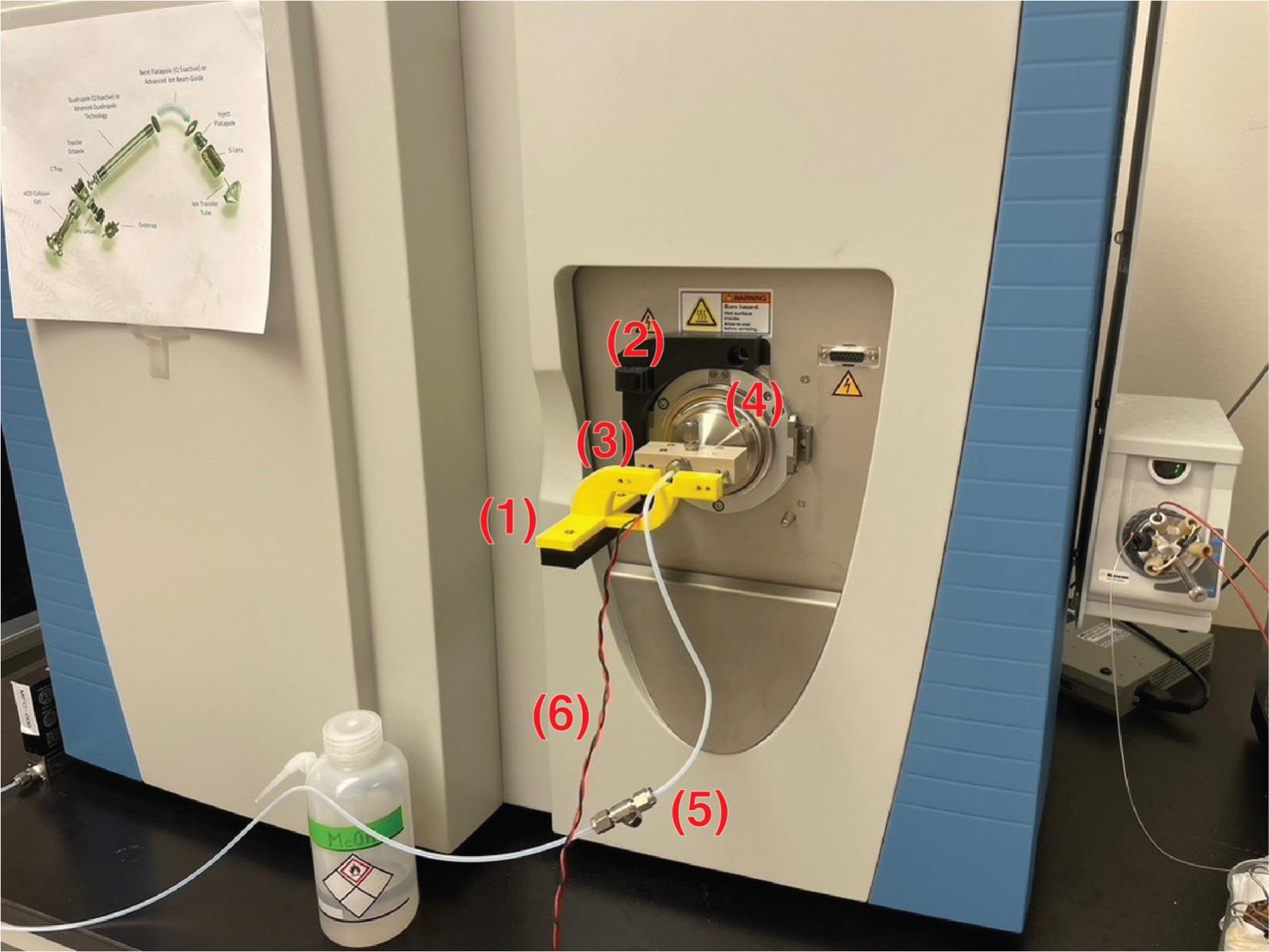


Supplemental Figure 17: An experimental apparatus for testing the ZAPPI chip with an atmospheric pressure orbitrap mass spectrometer. (1) The yellow 3D printed adjustable fixture to position the ZAPPI chip in line with the mass spectrometer inlet, (2) an intermediate fixture spoofing a commercial ion source allowing the MS to operate with ZAPPI attached, (3) the tan ZAPPI fixture and chip assembly, (4) the metallic atmospheric pressure inlet cone, (5) carrier gas inlet line, (6) power wires.


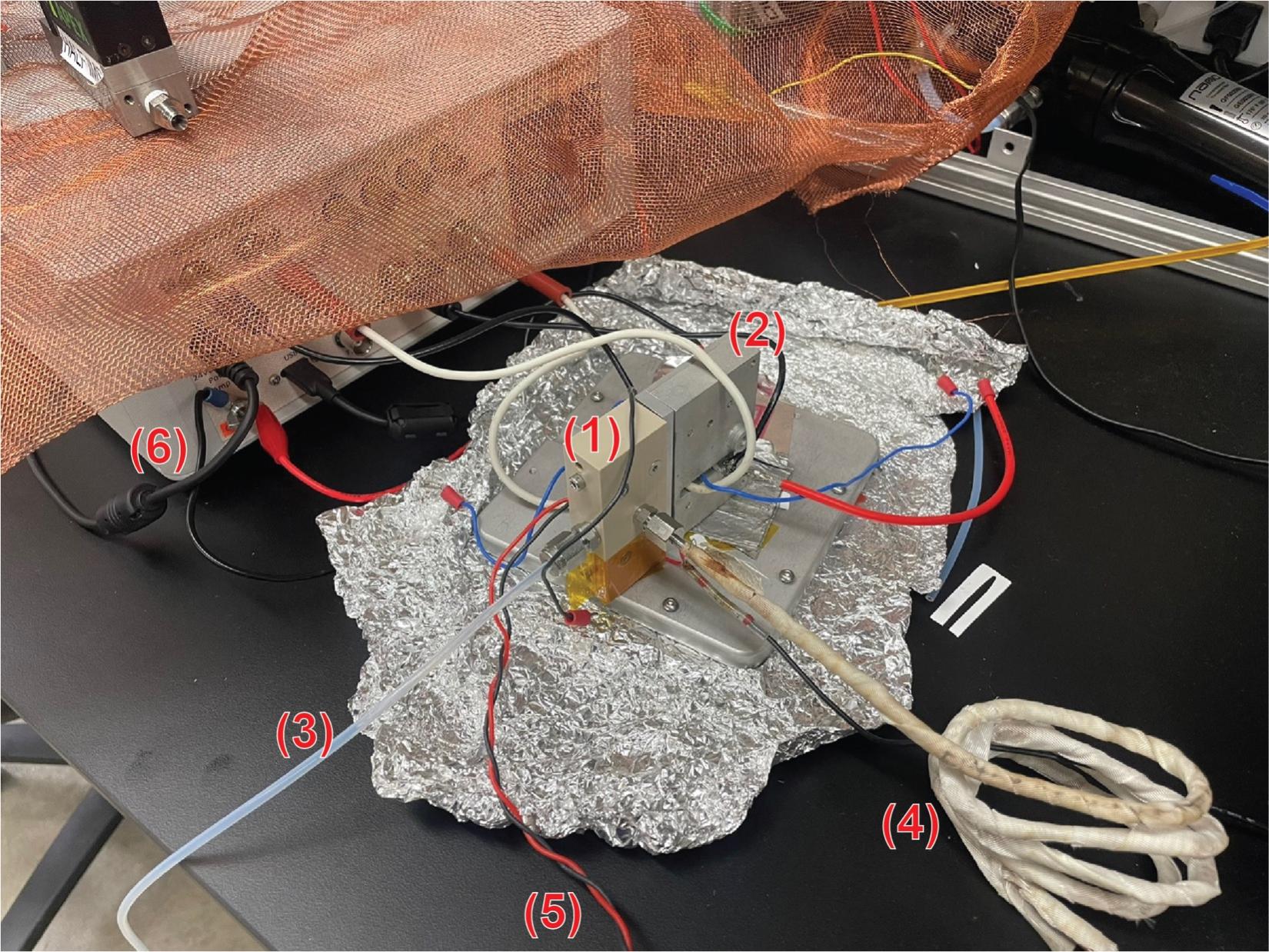


Supplemental Figure 18: Photograph of the faraday plate detector setup. (1) ZAPPI chip and PEEK fixture, (2) faraday plate chip and chip fixture, (3) carrier gas supply line, (4) heated analyte sample line, (5) power wires, (6) electrometer.
